# Utility of vertebral left atrial size and vertebral heart size to aid detection of congestive heart failure in dogs with respiratory signs

**DOI:** 10.1111/jvim.16918

**Published:** 2023-10-26

**Authors:** Evan S. Ross, Lance C. Visser, Nicholas Sbardellati, Brianna M. Potter, Alex Ohlendorf, Brian A. Scansen

**Affiliations:** ^1^ Department of Clinical Sciences College of Veterinary Medicine and Biomedical Sciences, Colorado State University Fort Collins Colorado USA; ^2^ Department of Environmental and Radiological Health Sciences College of Veterinary Medicine and Biomedical Sciences, Colorado State University Fort Collins Colorado USA

**Keywords:** canine, dyspnea, pulmonary edema, pulmonary hypertension, respiratory distress

## Abstract

**Background:**

Differentiating cardiogenic vs noncardiogenic causes of respiratory signs can be challenging when echocardiography is unavailable. Radiographic vertebral left atrial size (VLAS) and vertebral heart size (VHS) have been shown to predict echocardiographic left heart size, with VLAS specifically estimating left atrial size.

**Hypothesis/Objectives:**

Compare the diagnostic accuracy of VLAS and VHS to predict left‐sided congestive heart failure (CHF) in dogs presenting with respiratory signs.

**Animals:**

One‐hundred fourteen dogs with respiratory signs and radiographic pulmonary abnormalities.

**Methods:**

Retrospective cross‐sectional study. Dogs had to have an echocardiogram and thoracic radiographs obtained within 24 hours. Diagnosis of CHF was confirmed based on the presence of respiratory signs, cardiac disease, LA enlargement, and cardiogenic pulmonary edema.

**Results:**

Fifty‐seven dogs had CHF and 57 did not have CHF. Compared to VHS (area under the curve [AUC] 0.85; 95% confidence interval [CI], 0.77‐0.91), VLAS was a significantly (*P* = .03) more accurate predictor of CHF (AUC, 0.92; 95% CI, 0.85‐0.96). Optimal cutoff for VLAS was >2.3 vertebrae (sensitivity, 93.0%; specificity, 82.5%). Murmur grade (*P* = .02) and VLAS (*P* < .0001) were independently associated with CHF and VHS was not. Increased VHS (54%) was significantly (*P* = .01) more common than increased VLAS (24%) in dogs without CHF. Results were similar in a subsample of older and smaller dogs.

**Conclusions and Clinical Importance:**

When echocardiography is unavailable, VLAS and murmur grade have clinically utility to aid in differentiating cardiogenic from noncardiogenic respiratory signs. These findings might be especially useful to help rule out CHF in dogs with increased VHS that present with respiratory signs.

**Abbreviations**
AUC: area under the curve
CHF: congestive heart failure
EchoLAE: echocardiographic left atrial enlargement
LA: left atrial
LA/Ao_Sx: short‐axis left atrial dimension indexed to the short‐axis aortic diameter
LAD/AoD_Lx: long‐axis left atrial dimension indexed to the long‐axis aortic diameter
MMVD: myxomatous mitral valve disease
PH: pulmonary hypertension
VHS: vertebral heart size
VLAS: vertebral left atrial size

## 1 INTRODUCTION

Differentiating cardiogenic from noncardiogenic causes of respiratory signs in dogs is challenging, particularly if immediate access to or expertise in echocardiography is not available to document severe cardiac disease. Clinical signs of left‐sided congestive heart failure (CHF; eg, tachypnea, dyspnea, syncope) are also present in animals with noncardiac conditions such as respiratory disease or precapillary pulmonary hypertension (PH). Additionally, older dogs with cardiogenic respiratory signs secondary to myxomatous mitral valve disease (MMVD) would be expected to have a heart murmur but many dogs with noncardiogenic respiratory signs also might have a heart murmur because of the high prevalence of MMVD in this population. Recognition of cardiogenic vs noncardiogenic causes of respiratory signs can help optimize management and outcome.

A key determinant for differentiating cardiogenic from noncardiogenic respiratory signs is the presence of advanced heart disease determined by echocardiography performed by a skilled and knowledgeable operator, but is not always feasible particularly in an emergency setting. Thoracic radiography serves as a readily available alternative to help identify cardiomegaly as well as document pulmonary pathology. Recently, quantitative radiographic measurements of heart size, vertebral heart size (VHS)^1^ and vertebral left atrial size (VLAS),^2^ have been evaluated for their utility to predict clinically relevant cardiomegaly and therefore to aid in the staging and treatment of dogs with subclinical MMVD. Multiple studies have demonstrated that these measurements can serve as useful surrogates for echocardiographic measurements of left atrial (LA) and left ventricular size when echocardiography is not feasible^3^, ^4^, ^5^, ^6^, ^7^ and have been endorsed by American College of Veterinary Internal Medicine (ACVIM) expert guidelines for this purpose.^8^

Left atrial enlargement is a commonly used clinical surrogate for chronic and severely increased LA pressure. It is usually a critical confirmatory finding for dogs with suspected cardiogenic pulmonary edema showing respiratory signs. A recent study showed that VLAS was more accurate at detecting echocardiographic LA enlargement compared to VHS but not radiologists' interpretation of LA enlargement (yes/no) in 183 dogs with known or suspected cardiovascular disease.^3^ Qualitative assessment of radiographic LA size might be inaccurate, particularly with less experienced clinicians. Also, expert assessment by a radiologist might not be readily available.

The ability of VHS and VLAS to discriminate between cardiogenic vs noncardiogenic causes of respiratory signs in dogs is unknown but could prove clinically useful. Because VLAS has been shown to be a more accurate estimate of echocardiographically‐measured LA size compared to VHS,^3^ we hypothesize VLAS would show superior discriminatory ability to detect CHF within a cohort of dogs presenting with respiratory signs. Vertebral heart size is not specific to left heart size per se and can be influenced by additional factors such as right heart size. Our objective was to compare the diagnostic accuracy of VLAS and VHS to differentiate cardiogenic vs noncardiogenic causes of respiratory signs in dogs.

## 2 MATERIALS AND METHODS

In this retrospective study, records were searched between July 2020 and June 2022 for dogs that presented to our hospital for clinical signs suggestive of severe cardiac or respiratory disease (ie, “respiratory signs”). These signs included tachypnea with at least 1 of the following: dyspnea, acute onset or worsening of cough, or syncope. Tachypnea was defined as a respiratory rate >30 breaths/min if reported by the owner while their dog was sleeping at home^9^, ^10^, ^11^ or >41 breaths/min when obtained from a physical examination in the hospital.^12^ Dogs were eligible for inclusion if they had an echocardiographic examination and thoracic radiographs performed (consisting of at least 2 orthogonal views) within 24 hours of each other during the same hospital visit. Thoracic radiographs had to have evidence of pulmonary changes that potentially could be considered a manifestation of pulmonary edema. Echocardiographic examinations had to include standard imaging planes.^13^ Dogs were excluded if only a focused or brief echocardiogram was performed or if a right lateral thoracic radiograph projection was not acquired. Dogs also were excluded if thoracic radiographs indicated overt malpositioning of the patient, thoracic vertebral abnormalities (eg, hemivertebrae), or permitted limited visualization of the cardiac silhouette necessary for measurement of VLAS and VHS because of severe pulmonary pathology, a pulmonary mass, or pleural effusion. Dogs considered to have more than mild pericardial effusion or a cardiac mass (diagnosed by echocardiography) also were excluded.

All digital thoracic radiographs were reviewed and measured by the same study investigator (ESR) who was masked to the signalment and clinical information including clinical diagnosis, the diagnostic imaging service's assessment, and echocardiographic examination findings at the time of measurement. This investigator was a cardiology resident with extensive training and experience in performing cardiac measurements. The VLAS^2^ and VHS^1^ were measured from the right lateral projection using a digital caliper (Philips IntelliSpace Radiology—Enterprise, Philips Healthcare, Andover, MA). For VLAS, a line was drawn from the central and ventral border of the carina to the most caudal aspect of the left atrium at the intersection of the dorsal border of the caudal vena cava. A second line, equal in length, was indexed to thoracic vertebral bodies starting at the cranial edge of the fourth thoracic vertebrae (T4). For VHS, a line was extended from the same location of the carina to the farthest distance of the apex of the cardiac silhouette. A second line was drawn perpendicular to this line within the central 1/3 of the cardiac silhouette at its maximum width. The lengths of these 2 lines also were indexed to the thoracic vertebrae starting at T4 in an identical fashion to VLAS and summed. Both VLAS and VHS were measured to the nearest 0.1 vertebra. “Increased VLAS” was defined using published breed‐specific reference intervals,^14^, ^15^, ^16^, ^17^ if available. If unavailable, VLAS >2.2 vertebrae defined increased VLAS.^18^ “Increased VHS” was defined using published breed‐specific reference intervals,^14^, ^15^, ^19^, ^20^, ^21^, ^22^ if available. If unavailable, VHS >10.7 vertebrae defined increased VHS.^1^ See Table S1 for breed‐specific cutoffs utilized.

All echocardiographic examinations were performed by a board‐certified cardiologist or a supervised cardiology resident within our hospital's Cardiology & Cardiac Surgery Service. Echocardiograms were reviewed and remeasured for study purposes by a single investigator (ESR) at a digital off‐cart workstation (TomTec Imaging Systems GmbH, Chicago, IL). This investigator was blinded to the radiographic assessments at the time of measurement. When possible, measurements were performed on 3 consecutive cardiac cycles during sinus rhythm and averaged. Echocardiographic LA size was determined by 2 different techniques: (1) the short‐axis LA dimension indexed to the short‐axis aortic root (LA/Ao_Sx) and (2) the long‐axis LA dimension indexed to the long‐axis aortic diameter at the level of the aortic valve hinge points (LAD/AoD_Lx).^23^ Maximum long‐axis LA dimension was determined from a right parasternal long‐axis 4‐chamber view at end‐systole just before mitral valve opening. A line was drawn from the mid‐atrial septum to the internal reflection of the bright pericardium in the far field, approximately parallel to the mitral annulus. The diameter of the aorta in long‐axis was determined from a right parasternal long‐axis view optimized for the left ventricular outflow tract measured during early systole between the aortic valve hinge points. The LA/Ao_Sx was measured from the right parasternal short‐axis basilar view in early diastole, just after aortic valve closure using an inner edge‐to‐inner edge technique. Echocardiographic LA enlargement (EchoLAE) was defined by LA/Ao_Sx >1.68 and LAD/AoD_Lx >2.54.^23^ If a dog had an abnormality of the left ventricular outflow tract (eg, subaortic stenosis), EchoLAE was defined by maximum LA volume (right parasternal long‐axis 4‐chamber view using Simpson's method of discs) indexed to body weight (kg) >1.62 (mL/kg).^23^

For study purposes, dogs were grouped according to a diagnosis of CHF (yes/no) based on the consensus of 2 study investigators (ESR and LCV) after reviewing all clinical data that included a report reviewed by a board‐certified radiologist. Assessment for CHF was based on the aforementioned clinical signs necessary for study enrollment, the presence of cardiac disease with LA/Ao_Sx >1.68, LAD/AoD_Lx >2.54, or both, and radiographic evidence of cardiogenic pulmonary edema. For study purposes, precapillary PH was defined by a lack of EchoLAE, a tricuspid regurgitation velocity >3.4 m/s in the absence of a right ventricular outflow tract obstruction, and an intermediate or high probability of PH according to ACVIM guidelines.^24^ A subgroup analysis was performed of dogs ≥6 years of age and ≤20 kg.

### 2.1 Statistical analysis

Statistical tests were performed using commercial software (MedCalc Statistical Software, MedCalc Software bvba, Ostend, Belgium). Normality testing for continuous data was performed using the Shapiro‐Wilk test. Between group comparisons were performed using an unpaired *t*‐test or a Mann‐Whitney test based on normality testing. Ordinal data was compared using a Mann‐Whitney test. Continuous data were presented as median (25th and 75th percentiles). Categorical data were summarized as proportions, and percentages were compared with a chi‐squared test. Receiver operator characteristic curve analyses were performed to assess diagnostic accuracy, determine sensitivity and specificity percentages, and generate clinically relevant cut‐offs and likelihood ratios (LR) for VLAS and VHS to predict CHF (yes/no). DeLong's method was used for receiver operator characteristic curve comparison.^25^ After verifying that assumptions for logistic regression were met, univariable logistic regression analyses were performed to quantify the strength of association (odds ratios) of VHS, VLAS, and murmur grade with CHF. To identify which of these variables were independently associated with CHF, variables with *P* < .05 in the univariable model were entered into the multiple logistic model using a backwards stepwise method. Statistical significance was set at *P* < .05.

## 3 RESULTS

### 3.1 All study dogs

One hundred twenty‐one dogs were eligible for enrollment based on the inclusion criteria. Seven dogs were excluded; 4 because of incomplete echocardiograms, 3 for vertebral abnormalities (2 English bulldogs, 1 Boston Terrier). One‐hundred fourteen dogs made up the study sample with 57 dogs experiencing CHF (CHF‐yes) and 57 dogs not meeting criteria for CHF (CHF‐no). The echocardiographic diagnoses of dogs with CHF‐yes were as follows: myxomatous mitral valve disease (n = 36), dilated cardiomyopathy (n = 11), patent ductus arteriosus (n = 7), mitral valve stenosis (n = 1), subaortic stenosis (n = 1), and arteriovenous malformation (n = 1). The echocardiographic diagnoses of dogs with CHF‐no were as follows: precapillary PH (n = 31; 1 of which had heartworm disease) or either echocardiographically unremarkable or mild subclinical MMVD (ACVIM Stage B1) with a low probability of PH (n = 26). Twenty‐one dogs were mixed breed; 16 were Chihuahuas; 6 were Dachshunds; 4 each were Cavalier King Charles Spaniels, Toy Poodles, Pugs, Yorkshire Terriers; 3 each were Shetland Sheep Dogs, Jack Russell Terriers, German Shepherds, Labrador Retrievers, and American Pitbull Terriers; 2 each were Miniature Poodles, Bichon Frises, German Shorthaired Pointers, Boxers, Papillons, Australian Shepherds, Maltese, West Highland White Terriers, and the remaining were a Blue Heeler, Havanese, Bull Terrier, Akbash, Irish Wolfhound, Border Collie, Schnauzer, Shih Tzu, Whippet, Shiba Inu, Italian Greyhound, Mastiff, Portuguese Water Dog, Anatolian Shepherd, Irish Setter, Dalmatian, Chesapeake Bay Retriever, French Bulldog, Doberman Pinscher, Miniature Pinscher, Pembroke Welsh Corgi, Pomeranian, Japanese Chin, and American Cocker Spaniel.

Selected clinical, echocardiographic, and radiographic data of all dogs with CHF‐yes and CHF‐no are summarized in Table 1. When compared to CHF‐no, dogs with CHF‐yes were younger, had louder heart murmurs, and had increased heart size based on all echocardiographic and radiographic measures. Groups were similar based on body weight, heart rate, and respiratory rate as well as clinical signs of dyspnea, acute onset or worsening cough, and syncope. The percentage of dogs with increased VLAS in the CHF‐no group was 24%, which was significantly different (*P* = .01) from the percentage of dogs that had increased VHS (54%). Increased VLAS and VHS were common in the CHF‐yes group at 95% and 93%, respectively.

**TABLE 1:** Selected clinical, echocardiographic, and radiographic data of 114 dogs with respiratory signs.

Variables	CHF‐no (n = 57)	CHF‐yes (n = 57)	*P*‐value
Age (years)	12.0 (10.0, 14.0)	10.0 (6.0, 13.0)	.002
Body weight (kg)	7.4 (4.0, 15.0)	9.0 (5.3, 24.0)	.24
Heart rate (min^−1^)	140 (120, 160)	144 (120, 163)	.59
Respiratory rate (min^−1^)	60 (43, 111)	60 (40, 100)	.64
Murmur grade (out of 6)	3 (0, 4)	5 (4, 5)	<.0001
Dyspnea: proportion (%)	41/57 (71.9%)	43/57 (75.4%)	.67
Syncope: proportion (%)	12/57 (21.0%)	14/57 (24.6%)	.66
Cough: proportion (%)	20/57 (35.1%)	19/57 (33.3%)	.84
LAD/AoD_Lx^a^	2.48 (2.22, 2.84)	3.91 (3.59, 4.33)	<.0001
LA/Ao_Sx^a^	1.32 (1.22, 1.49)	2.24 (2.00, 2.58)	<.0001
EchoLAE: proportion (%)	7/57 (12.3%)	55/57 (96.5%)	<.0001
VLAS (vertebrae)	2.1 (1.9, 2.2)	3.0 (2.8, 3.2)	<.0001
Increased VLAS: proportion (%)	14/57 (24%)^b^	54/57 (95%)	<.0001
VHS (vertebrae)	11.1 (10.1, 11.8)	12.9 (11.9, 14.1)	<.0001
Increased VHS: proportion (%)	31/57 (54%)	53/57 (93%)	<.0001

*Note*: Data are presented median (IQR) unless stated otherwise.

Abbreviations: CHF, left‐sided congestive heart failure; EchoLAE, echocardiographic left atrial enlargement; LAD/AoD_Lx, left atrial dimension indexed to the aortic valve annulus in long‐axis; LA/Ao_Sx, left atrium to aortic root ratio in short‐axis; VLAS, vertebral left atrial size; VHS, vertebral heart size.

^a^ Not quantified in the 1 dog with subaortic stenosis.

^b^ Significantly different from the proportion of dogs with increased VHS in the CHF‐no group (*P* = .007).

**FIGURE 1:**
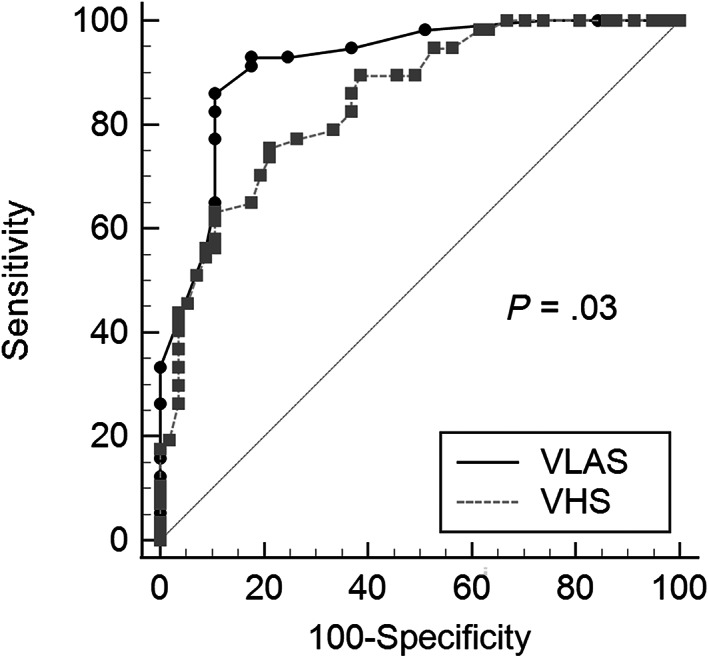
Comparison of the receiver operating characteristic curves for vertebral left atrial size (VLAS, solid black line) and vertebral heart size (VHS, dotted gray line) for detecting congestive heart failure in 114 dogs with respiratory signs.

Diagnostic accuracy, cut‐offs, and likelihood ratios of VLAS and VHS for predicting CHF are presented in Table 2. The diagnostic accuracy of VLAS (area under the curve [AUC], 0.92; 95% confidence interval [CI], 0.85‐0.96) to detect CHF was significantly (*P* = .03) higher than VHS (AUC, 0.85; 95% CI, 0.77‐0.91; Figure 1). The optimal cut‐off for the discriminatory ability of VLAS to detect CHF based on the highest Youden index was >2.3 vertebrae and for VHS was >11.8 vertebrae. Multiple logistic regression showed that VLAS (*P* < .0001) and murmur grade (*P* = .02) were independently associated with a diagnosis of CHF, whereas VHS was not (Table 3).

**TABLE 2:** Receiver operating characteristic curve analyses to determine the diagnostic accuracy of the radiographic measurements for detecting congestive heart failure in 114 dogs with respiratory signs.

Radiographic variables	AUC (95% CI)^a^	*P*‐value	Cutoff (vertebrae)	Cutoff type	Sn (%)	Sp (%)	+LR	−LR
VLAS	0.92 (0.85‐0.96)	<.0001	>2.3	Optimal	93.0	82.5	5.30	0.09
			>1.9	Max Sn	100	26.3	1.36	0.00
			>3.0	Max Sp	33.3	100	n/a	0.67
VHS	0.85 (0.77‐0.91)	<.0001	>11.8	Optimal	75.4	79.0	3.58	0.31
			>10.6	Max Sn	100	33.3	1.50	0.00
			>14.4	Max Sp	17.4	100	n/a	0.82

Abbreviations: +LR, positive likelihood ratio; AUC, area under the curve; CI, confidence interval; −LR, negative likelihood ratio; Sn, sensitivity; Sp, specificity; VHS, vertebral heart size; VLAS, vertebral left atrial size.

^a^ Pairwise comparison of receiver operator characteristic curves was significantly different (*P* = .03).

**TABLE 3:** Logistic regression analyses to identify clinical and radiographic variables independently associated with congestive heart failure in 114 dogs with respiratory signs.

	Univariable logistic regression		Multiple logistic regression
Radiographic variables	Odds ratio (95% CI)	*P*‐value	*P*‐value
VLAS	96.1 (22.2‐415.8)	<.0001	<.0001
VHS	3.3 (2.1‐5.1)	<.0001	–
Murmur grade	1.9 (1.5‐2.5)	<.0001	.02

*Note*: “–” = variable not included in the final model.

Abbreviations: CI, confidence interval; VHS, vertebral heart size; VLAS, vertebral left atrial size.

### 3.2 Dogs ≤20 kg and ≥6 years old

Eighty‐one dogs made up a subsample representing older and smaller dogs; 34 CHF‐yes and 47 CHF‐no. The echocardiographic diagnoses of dogs with CHF‐yes were MMVD in 33 dogs and dilated cardiomyopathy in 1 dog. The echocardiographic diagnoses of dogs with CHF‐no were as follows: precapillary PH (n = 30) or either echocardiographically unremarkable or mild subclinical MMVD (ACVIM Stage B1) with a low probability of PH (n = 17).

Selected clinical, echocardiographic, and radiographic data of dogs with CHF‐yes and CHF‐no in this subsample are summarized in Table 4. When compared to CHF‐no dogs, dogs with CHF‐yes had louder heart murmurs, and had increased heart size based on all echocardiographic and radiographic measures. Both groups had similar age, body weight, heart rate, and respiratory rate as well as clinical signs of dyspnea, acute onset or worsening cough, and syncope. The percentage of dogs with increased VLAS in the CHF‐no group was 23%, which was significantly different (*P* = .04) from the percentage of dogs that had increased VHS (55%). Increased VLAS and VHS were common in CHF‐yes group at 91% and 88%, respectively.

**TABLE 4:** Selected clinical, echocardiographic, and radiographic data of 81 dogs ≤20 kg and ≥6 years old with respiratory signs.

Variables	CHF‐no (n = 47)	CHF‐yes (n = 34)	*P*‐value
Age (years)	12.0 (11.0, 14.0)	11.5 (10.0, 13.0)	.16
Body weight (kg)	6.5 (3.7, 9.2)	7.0 (5.0, 10.7)	.50
Heart rate (min^−1^)	144 (130, 160)	140 (120, 170)	.67
Respiratory rate (min^−1^)	60 (42, 111)	66 (40, 100)	.99
Murmur grade (out of 6)	3 (1, 4)	5 (4, 5)	<.0001
Dyspnea: proportion (%)	34/47 (72.3%)	25/34 (73.5%)	.91
Syncope: proportion (%)	11/47 (23.4%)	11/34 (32.4%)	.37
Cough: proportion (%)	16/47 (34.0%)	17/34 (50.0%)	.15
LAD/AoD_Lx	2.60 (2.36, 2.89)	4.05 (3.63, 4.40)	<.0001
LA/Ao_Sx	1.32 (1.22, 1.48)	2.27 (2.00, 2.63)	<.0001
LA enlargement: proportion (%)	6/47 (12.8%)	34/34 (100%)	<.0001
VLAS (vertebrae)	2.0 (1.9, 2.2)	3.0 (2.8, 3.0)	<.0001
Increased VLAS: proportion (%)	11/47 (23%)^a^	31/34 (91%)	<.0001
VHS (vertebrae)	10.2 (7.0, 11.1)	12.6 (11.5, 13.8)	<.0001
Increased VHS: proportion (%)	26/47 (55%)	30/34 (88%)	.002

*Note*: Data are presented median (IQR) unless stated otherwise.

Abbreviations: CHF, left‐sided congestive heart failure; LA/Ao_Sx, left atrium to aortic root ratio in short‐axis; LAD/AoD_Lx, left atrial dimension indexed to the aortic valve annulus in long‐axis; VHS, vertebral heart size; VLAS, vertebral left atrial size.

^a^ Significantly different from the proportion of dogs with increased VHS in the CHF‐no group (*P* = .04).

**FIGURE 2:**
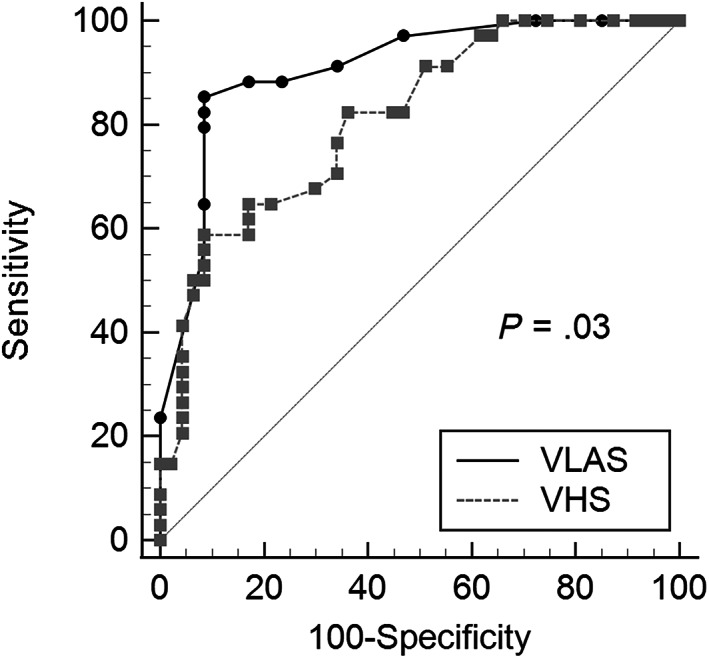
Comparison of the receiver operating characteristic curves for vertebral left atrial size (VLAS, solid black line) and vertebral heart size (VHS, dotted gray line) for detecting congestive heart failure in a subsample of 81 dogs ≤20 kg and ≥6 years old with respiratory signs.

Diagnostic accuracy, cut‐offs, and likelihood ratios of VLAS and VHS for detecting CHF in this subsample are presented in Table 5. The diagnostic accuracy of VLAS (AUC, 0.91; 95% CI, 0.82‐0.96) for detecting CHF was significantly (*P* = .03) higher than VHS (AUC, 0.81; 95% CI, 0.71‐0.89; Figure 2). The optimal cut‐off for VLAS detecting CHF‐yes based on the highest Youden index was >2.5 vertebrae and for VHS was >12.2 vertebrae. Multiple logistic regression showed that VLAS (*P* < .0001) and murmur grade (*P* = .03) were independently associated with CHF, whereas VHS was not (Table 6).

**TABLE 5:** Receiver operating characteristic curve analyses to determine the diagnostic accuracy of the radiographic measurements for detecting congestive heart failure in 81 dogs ≤20 kg and ≥6 years old with respiratory signs.

Radiographic variables	AUC (95% CI)^a^	*P*‐value	Cutoff (vertebrae)	Cutoff type	Sn (%)	Sp (%)	+LR	−LR
VLAS	0.91 (0.82‐0.96)	<.0001	>2.5	Optimal	85.3	91.5	10.02	0.16
			>1.9	Max Sn	100	27.7	1.39	0.00
			>3.0	Max Sp	23.5	100	n/a	0.76
VHS	0.81 (0.71‐0.89)	<.0001	>12.2	Optimal	58.8	91.5	6.91	0.45
			>10.6	Max Sn	100	34.0	1.52	0.00
			>14.4	Max Sp	14.7	100	n/a	0.85

Abbreviations: AUC, area under the curve; CI, confidence interval; −LR, negative likelihood ratio; +LR, positive likelihood ratio; Sn, sensitivity; Sp, specificity; VLAS, vertebral left atrial size; VHS, vertebral heart size.

^a^ Pairwise comparison of receiver operator characteristic curves was significantly different (*P* = .03).

**TABLE 6:** Logistic regression analyses to identify clinical and radiographic variables independently associated with congestive heart failure in 81 dogs ≤20 kg and ≥6 years old with clinical signs of cardiopulmonary disease.

	Univariable logistic regression		Multiple logistic regression
Radiographic variables	Odds ratio (95% CI)	*P*‐value	*P*‐value
VLAS	77.5 (15.0‐399.6)	<.0001	<.0001
VHS	2.8 (1.7‐4.6)	<.0001	–
Murmur grade	2.9 (1.7‐5.0)	.03	.03

*Note*: “–” = variable not included in the final model.

Abbreviations: CI, confidence interval; VHS, vertebral heart size; VLAS, vertebral left atrial size.

## 4 DISCUSSION

Our results supported the hypothesis that, when compared to VHS, VLAS is better at predicting the presence or absence of CHF in dogs presenting with respiratory signs. When compared to VHS, murmur grade and VLAS were independently associated with CHF. When results were analyzed in a subsample of older (≥6 years) and smaller dogs (≤20 kg) where CHF secondary to MMVD is a common presumptive diagnosis, nearly identical findings were apparent. Optimal cut‐offs for VLAS and VHS were slightly higher in this subsample at >2.5 and >12.2, respectively. Diagnostic accuracy for VHS (AUC, 0.81; 95% CI, 0.71‐0.89) exhibited suboptimal clinical utility (AUC <0.85) in this context.^26^

Unlike previous studies evaluating the discriminatory ability of VHS, VLAS, or both to help identify clinically important cardiac chamber enlargement,^2^, ^3^, ^4^, ^5^, ^6^, ^7^, ^27^, ^28^, ^29^ we evaluated the ability of VLAS and VHS to support or refute a cardiogenic cause for respiratory signs in a clinical setting and in a sample of dogs not solely affected by MMVD. Thus, our results bear more direct relevance to the clinical management of dogs with respiratory signs. Our results suggest that, in a dog showing respiratory signs and radiographic pulmonary changes where echocardiography is not feasible, a VLAS >2.3 vertebrae suggests the respiratory signs are cardiogenic in origin. Conversely, if the VLAS is ≤2.3 vertebrae, our results suggest this decreases the likelihood of cardiogenic respiratory signs (sensitivity was 93%). Results were slightly different when confined to older (≥6 years) and smaller (≤20 kg) dogs where CHF secondary to MMVD is a common presumptive diagnosis in dogs with a heart murmur and respiratory signs. We performed this subanalysis to determine if results differed substantially from the total study sample. We anticipated this information would be useful for practitioners who understandably presume dogs of this signalment that have heart murmurs are affected with MMVD. In this context, results suggest a VLAS >2.5 vertebrae markedly increases the likelihood that a dog has cardiogenic respiratory signs (specificity was 92%). If VLAS is ≤2.5, our results suggest respiratory signs are unlikely to be cardiogenic in this subsample. Regardless of the signalment, if VLAS approaches values of ≤2.0 vertebrae, clinicians can more confidently rule out cardiogenic respiratory signs. As VLAS approaches values of ≥3.0, clinicians can be confident of cardiogenic respiratory signs. These findings, when viewed within the clinical context, might help focus initial treatment such as prompting empirical administration of furosemide or justify additional furosemide dosages for CHF, vs alternative treatments tailored to noncardiac diseases.

Our study demonstrates that VLAS has superior diagnostic accuracy for the specific purpose of differentiating cardiogenic vs noncardiogenic respiratory signs relative to VHS. Our study design differs from previous studies that evaluated the diagnostic accuracy of VHS and VLAS for predicting clinically important cardiac chamber enlargement.^3^, ^4^, ^5^, ^7^, ^27^, ^28^, ^29^ These studies primarily evaluated dogs with subclinical cardiac disease and most evaluated only dogs with subclinical MMVD. Our study included a more diverse sample of dogs with clinical signs. Our results agree with a previous study^3^ that also found VLAS exhibited superior diagnostic accuracy compared to VHS for detection of echocardiographic LA enlargement in a large and diverse sample of dogs with known and suspected cardiovascular disease with and without echocardiographic LA enlargement. Another study found that VLAS marginally outperformed VHS for prediction of normal echocardiographic heart size in large breed dogs.^27^

We conclude that VLAS is a better predictor of CHF compared to VHS. Our study demonstrates that many dogs with respiratory signs and pulmonary changes that are not attributable to CHF have cardiomegaly when defined by increased VHS—54% in our study, whereas increased VLAS was present in a significantly lower percentage of dogs (24%) in the CHF‐no group. This result is likely because VLAS is a radiographic measurement intended to be more specific to LA size. Conversely, VHS quantitates the length and width of the cardiac silhouette and can be influenced by other factors such as enlargement of the right heart, respiratory and cardiac cycles,^30^, ^31^ and ventricular wall thickness. Although not specifically evaluated in our study, right heart enlargement would not be surprising in dogs with noncardiogenic respiratory signs, because 31 dogs (54%) had precapillary PH.^32^, ^33^, ^34^

Our study evaluated 2 logistic regression models, 1 from the entire study sample and 1 from a subsample of older small dogs where a presumptive diagnosis of MMVD is likely to be common in a dog presenting with respiratory signs and a heart murmur. Each model evaluated if murmur grade, VHS, or VLAS was independently associated with CHF. Murmur grade was added to the model along with VLAS and VHS because if it was identified as an independent predictor of CHF, clinicians could deduce that a lack of a murmur or a soft murmur makes CHF less likely and louder murmurs make CHF more likely in patients presenting with respiratory signs. Our results showed that both murmur grade and VLAS were positively and independently associated with CHF. This conclusion held true for the entire study sample as well as the subsample of older small dogs. Clinicians should not overlook that murmur grade (or lack of a murmur) might be helpful when attempting to rule in or rule out CHF in a dog presenting with respiratory signs.

Our results should be interpreted within the context of the retrospective study design and its limitations. The interpretation of our results largely hinges upon the grouping of CHF‐yes vs CHF‐no. A definitive assessment of whether a dog is experiencing CHF can be challenging in some cases and is ultimately a matter of opinion. We acknowledge that misdiagnoses of CHF are possible in our study and could have impacted the diagnostic accuracy of VHS and VLAS. Opinions regarding the presence of CHF can vary but should involve the assimilation of all clinical information available to the clinician and be based on a multifactorial approach. We believe VLAS can serve as a helpful component within a multifactorial approach for the assessment of CHF in dogs, particularly when echocardiography is not feasible.

Both VHS and VLAS have inherent limitations and can be challenging to measure or are open to interpretation, particularly with poor visualization of the carina or borders of the cardiac silhouette. Our results only apply to dogs with respiratory signs where VHS and VLAS can be measured, which is not always possible in practice. Based on the retrospective study design, medications with effects on the cardiovascular system were not standardized and could have been administered between the radiographic and echocardiographic examinations. However, these effects should have impacted our cardiac measurements equally. We also acknowledge that having a single investigator perform measurements could be considered a limitation. We did so to maintain consistency in how the measurements were performed. However, this approach could limit the external validity of our results, because a single trained investigator does not represent all individuals (with variable levels of expertise and experience) performing radiographic measurements for clinical purposes. Multiple previous studies have demonstrated that measurement variability of VHS, VLAS, or both are relatively stable when comparing different investigators.^2^, ^4^, ^6^, ^18^, ^35^, ^36^

In conclusion, when echocardiography is unavailable, VLAS and murmur grade have clinical utility to aid in differentiating cardiogenic from noncardiogenic causes of respiratory signs, whereas VHS might be less useful. These results might be especially useful to help rule out CHF in dogs with respiratory signs and increased VHS.

## CONFLICT OF INTEREST DECLARATION

Authors declare no conflict of interest.

## OFF‐LABEL ANTIMICROBIAL DECLARATION

Authors declare no off‐label use of antimicrobials.

## INSTITUTIONAL ANIMAL CARE AND USE COMMITTEE (IACUC) OR OTHER APPROVAL DECLARATION

Authors declare no IACUC or other approval was needed.

## HUMAN ETHICS APPROVAL DECLARATION

Authors declare human ethics approval was not needed for this study.

## Supporting information


**Table S1.** Published breed‐specific cutoffs used to define increased vertebral heart size and vertebral left atrial size for the dogs enrolled in this study.
