# Supplementary material for: Utility of vertebral left atrial size and vertebral heart size to aid detection of congestive heart failure in dogs with respiratory signs
Source: J Vet Intern Med. 2023 Oct 26;37(6):2021–9. doi: 10.1111/jvim.16918 (PMC10658574; doi:10.1111/jvim.16918)
Supplement: Supplementary file 1 — Table S1. Published breed‐specific cutoffs used to define increased vertebral heart size and vertebral left atrial size for the dogs enrolled in this study. [file JVIM-37-2021-s001.pdf]

Supplemental Table

SUPPLEMENTAL TABLE. Published breed-specific cutoffs used to define increased vertebral heart size and vertebral left atrial size for the dogs enrolled in this study.

Radiographic measurement	Breed	Cutoff (vertebrae)*	Reference
Vertebral heart size	Mixed	>10.7	Buchanan 1995
	Chihuahua	>11.2	Puccinelli 2021
	Dachshund	>10.7	Jepsen-Grant 2013
	Cavalier King Charles Spaniel	>11.6	Lamb 2001
	Pug	>12.5	Jepsen-Grant 2013
	Yorkshire Terrier	>11.1	Jepsen-Grant 2013
	German Shepherd	>11.3	Lamb 2001
	Labrador Retriever	>10.8	Bodh 2016
	Boxer	>13.2	Lamb 2001
	Whippet	>12.0	Bavegems 2005
	Doberman Pinscher	>11.2	Lamb 2001
	Pomeranian	>12.3	Jepsen-Grant 2013
	Maltese	>10.5	Baisan 2022
	Shih Tzu	>10.7	Jepsen-Grant 2013
Vertebral left atrial size	Mixed	>2.2	Vezzosi 2020
	Chihuahua	>2.1	Puccinelli 2021

Cavalier King Charles Spaniel	>1.9	Bagardi 2022
Pug	>2.8	Wiegel 2022
Maltese	>2.3	Baisan 2022

*Cutoff determined as mean + 2 × standard deviation to define the upper limit for the reference interval.
